# Leptomeningeal Carcinomatosis With Optic Nerve Metastasis Secondary to Breast Cancer

**DOI:** 10.7759/cureus.14200

**Published:** 2021-03-31

**Authors:** Chiachee Chew, Wan-Hazabbah Wan Hitam, Liza Sharmini Ahmad Tajudin

**Affiliations:** 1 Department of Ophthalmology and Visual Science, School of Medical Sciences, Universiti Sains Malaysia, Kubang Kerian, MYS

**Keywords:** leptomeningeal carcinomatosis, carcinomatous meningitis, optic nerve infiltration, breast cancer

## Abstract

Leptomeningeal carcinomatosis (LC) and optic nerve metastasis are uncommon occurrences in breast cancer. We report a rare case of LC with optic nerve infiltration secondary to breast cancer. A 45-year-old lady who was a known case of treated right breast carcinoma six years ago presented with a blurring of vision in both eyes, floaters, and diplopia for one month. She also had recurrent attacks of seizure-like episodes, headache, and vomiting. Examination revealed high blood pressure with tachycardia. Her right eye visual acuity was counting fingers at two feet and 6/36 in the left eye. She had right abducens nerve palsy. Fundoscopy showed bilateral optic disc swelling with pre-retinal, flame-shaped haemorrhages and macular oedema. CT scan of brain and orbit was normal. She was admitted for further investigations. While in the ward, her vision deteriorated further. Her visual acuity in both eyes was at the level of no perception to light. She also developed bilateral abducens nerve palsy and right facial nerve palsy. Subsequently, she started having bilateral hearing loss. There were few episodes of fluctuations in conscious awareness. MRI brain showed mild hydrocephalus. Both optic nerves were thickened and enhanced on T1-weighted and post-gadolinium. Lumbar puncture was performed. There was high opening pressure. Cerebrospinal fluid cytology showed the presence of malignant cells. Family members opted for palliative care in view of poor prognosis. Unfortunately, she succumbed after a month's stay in hospital. Diagnosis of LC and optic nerve infiltration presents a formidable challenge to clinicians especially in the early stages where neuroimaging appears normal and lumbar puncture has high false negatives. Multiple high-volume taps are advised if clinical suspicion of LC is high.

## Introduction

Solid tumours can metastasize to the central nervous system in two different ways: cerebral metastases or leptomeningeal carcinomatosis (LC). LC is cancer involving pia mater and arachnoid matter and it is rare. Tumour cells reach the leptomeninges by hematogenous spread or by direct extension from pre-existing lesions. Most solid tumours are known to cause LC, and metastatic breast cancer is the most common cancer reported to result in LC followed by lung cancer and melanoma. Optic nerve metastasis is even rarer, with only 0.4% of breast cancer reported to infiltrate the optic nerves [1]. LC poses a diagnostic challenge due to asymptomatic or presence of unspecific symptoms that may not prompt evaluation in a sick patient and low sensitivity of different diagnostic modalities. LC carries a poor prognosis and limited treatment options. Treatment mainly focuses on improving the quality of life and provide supportive care. We report a rare case of LC with optic nerve infiltration secondary to breast cancer. 

## Case presentation

A 45-year-old premenopausal lady presented with a progressive blurring of vision in both eyes for one month. It began with seeing floaters and having double vision. She also had intermittent headaches, nausea, and vomiting for the past three months that were increasing in frequency and severity. She developed recurrent attacks of seizure-like episodes with a sudden loss of consciousness lasting for few minutes. It was associated with neck and arm stiffness. The episode resolved spontaneously.

She was diagnosed to have stage-2 right-breast carcinoma six years ago. Biopsy was performed and confirmed infiltrating lobular carcinoma in the private sector and she underwent a right mastectomy and axillary clearance. She was started on oral tamoxifen in view of positive for estrogen and progesterone receptor. However, she defaulted after two years of follow-up in view of financial constraints and claimed that she was well. She visited five years later when she felt a lump over her right axillary region. She was detected to have multiple enlarged right axillary lymph nodes on a mammogram. CT scan of thorax, abdomen, and pelvis (CT-TAP) revealed distant metastasis to the bone and staging was T4N2M1. She underwent a second surgery of right axillary clearance. Biopsy showed metastatic lobular carcinoma with luminal subtype. She completed six cycles of 5-fluorouracil, epirubicin, cyclophosphamide (FEC) chemotherapy, 15 cycles of radiotherapy, and was continued on tamoxifen.

**Figure 1:**
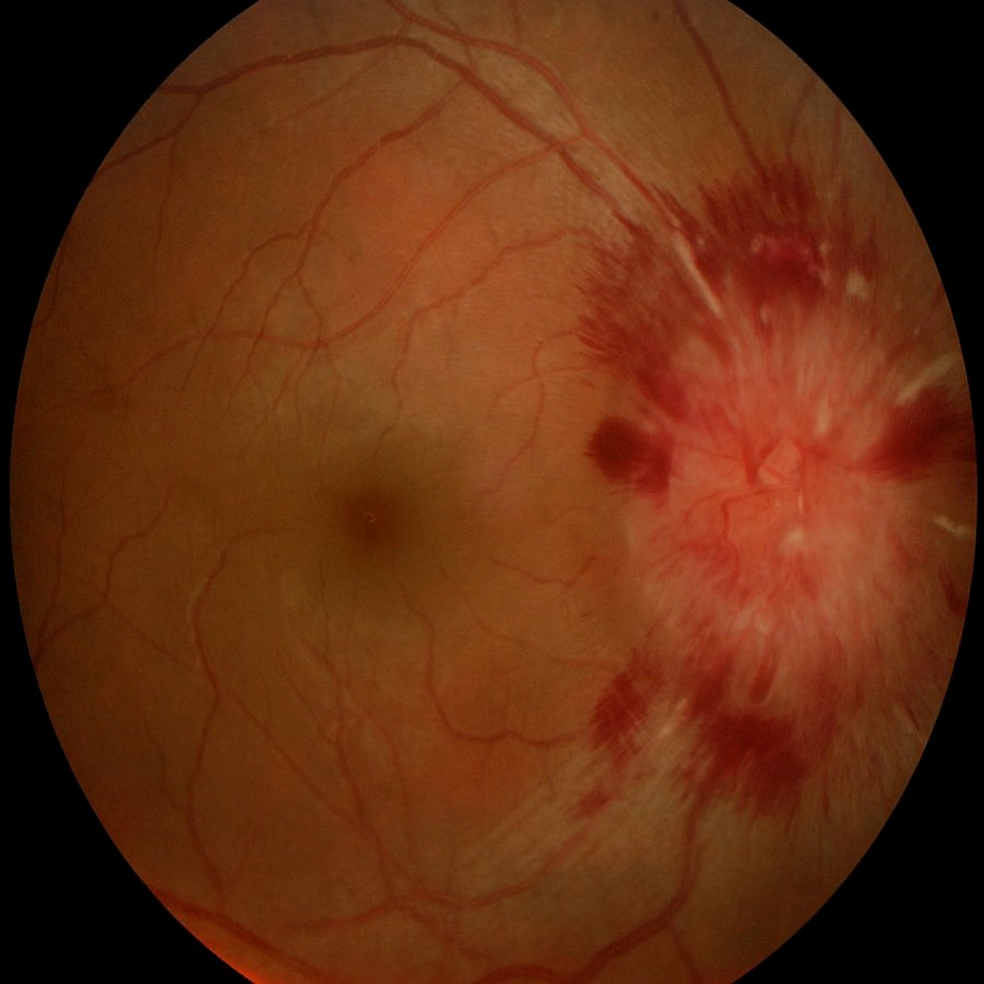
Funduscopy of right eye showing diffuse optic disc swelling with surrounding multiple flame-shaped hemorrhages and venous tortuosity.

**Figure 2:**
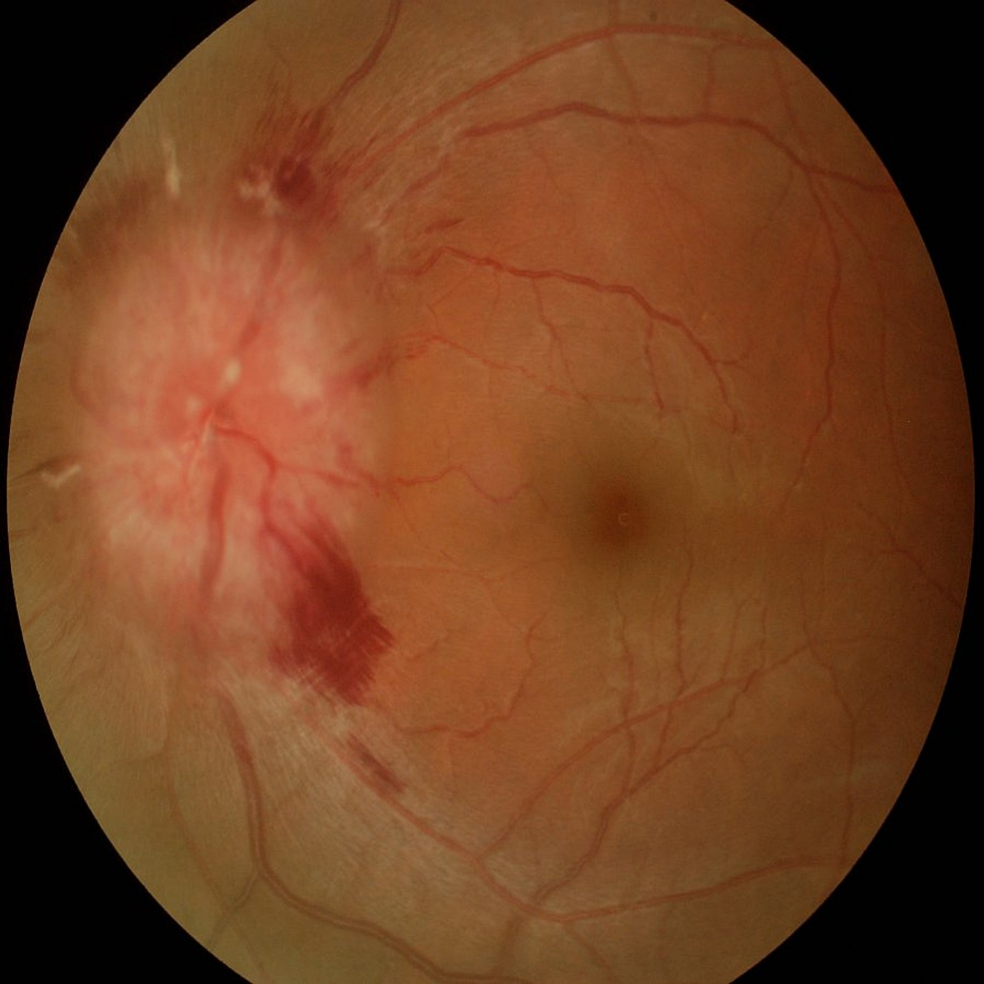
Fundus photo of left eye showing diffuse optic disc swelling with flame-shaped hemorrhage at superior and inferior temporal quadrant of disc margin, cotton wool spot at peripapillary region.

On systemic examination, her blood pressure was high (170/108 mmHg) with tachycardia (pulse rate 140 beats per minute). Electrocardiogram (ECG) showed sinus tachycardia. Ocular examination revealed her right eye visual acuity was 6/36 and counting fingers at 2 feet in the left eye. Optic nerve functions could not be assessed in view of the patient's reluctance. She had right abducens nerve palsy with limitation of abduction. Both pupils were poorly reactive to light. Fundoscopy showed bilateral optic disc swelling with pre-retinal and flame-shaped haemorrhages with venous tortuosity (Figures 1, 2). There was no choroidal lesion seen. CT Scan of brain and orbit on arrival was normal.

She was admitted for further investigations. While in the ward, her vision deteriorated further. Her visual acuity in both eyes was at the level of no perception to light in all four quadrants. She also developed bilateral abducens nerve palsy and right facial nerve palsy. Subsequently, she started having bilateral hearing loss. There were a few episodes of fluctuations in conscious awareness associated with high blood pressure.

**Figure 3:**
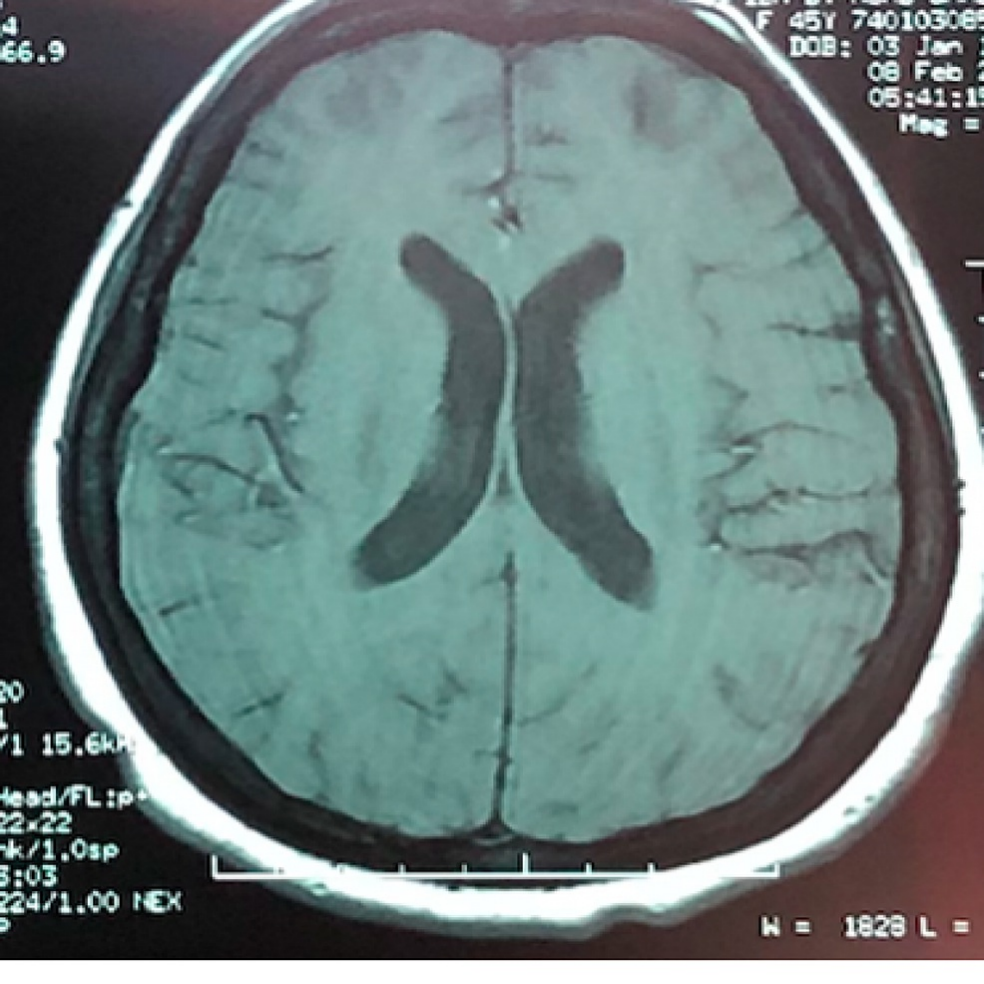
Plain MRI brain showing hydrocephalus.

**Figure 4:**
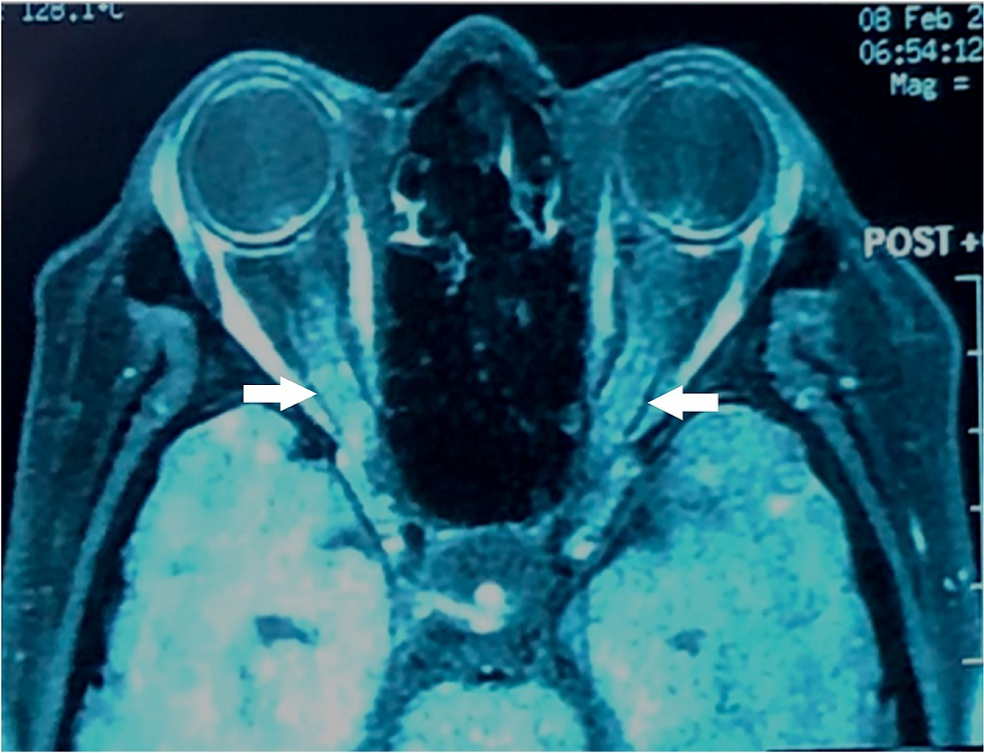
T1-weighted MRI orbit post-gadolinium showing generalised bilateral optic nerve thickening and enhancement (white arrows).

An MRI brain urgent performed two days after admission showed mild hydrocephalus (Figure 3). Both optic nerves were thickened and enhanced on T1-weighted and post-gadolinium (Figure 4). A lumbar puncture was performed. There was high opening pressure at 48 cmH20. Cerebrospinal fluid (CSF) cytology showed the presence of malignant cells. She was subsequently diagnosed with leptomeningeal carcinomatosis with optic nerve metastasis secondary to breast cancer. Multidisciplinary teams discussion involving oncologist, breast surgeon, ophthalmologist, and palliative care physician was done and planned for intrathecal chemotherapy. Family members opted for palliative care in view of poor prognosis and the patient being ill. Unfortunately, she succumbed after a month of staying in the ward.

## Discussion

Leptomeningeal carcinomatosis is the infiltration of the meninges from a solid primary tumour. It can involve the pia mater and arachnoid and subarachnoid spaces. It can spread via hematogenic, direct extension, along perineurial lymphatics, and CSF. It is a rare condition seen in 4-15% of patients with solid tumours [2]. It carries a poor prognosis and high mortality, with a median survival of 4-6 weeks if left untreated [3].

Although only 5% of breast cancer patients develop leptomeningeal metastases [4,5], it is still the most common etiology of LC [6]. Common symptoms at presentation are headache, cranial nerve palsies (mainly VI, VII, and VIII cranial nerves), dizziness, seizures, and signs of elevated intracranial pressure (ICP) [7], all of which were found in our patient.

Lumbar puncture with CSF analysis is known to carry high false negatives and thus multiple high-volume taps are recommended by many experts. Repeated CSF sampling of at least 10-20ml should be sent for cytology. CSF cytology has been reported to have a sensitivity of 75-77% in leptomeningeal metastases, but more than two examinations were needed in 72% of positive cases [8,9]. Flow cytometry is more sensitive than conventional cytology and can be performed if clinical suspicion of LC is high [10]. For our patient, 30 ml of CSF was obtained without effort during lumbar puncture due to raised intracranial pressure. Multiple sections of CSF cytological examination was performed to avoid false negative results.

Our patient also had optic nerve metastases. Most of the time, optic nerve involvement is bilateral. Loss of vision is the main symptom for breast cancer secondaries to the optic nerve infiltration [11]. Our patient presented with a gradual decrement in her visual acuity over a month which acutely ‘wiped out' within three days. Optic nerve metastasis by breast cancer usually occurs after treatment of the primary cancer and runs an indolent course. It can manifest as an isolated nodule over the optic nerve, but more commonly it diffusely infiltrates the entire optic nerve and causes a slight uniform thickening. The metastatic tumour can also show up as a diffuse enlargement of the optic nerve head or as a well-circumscribed optic nerve head mass [12]. In our case, enlargement of optic disc was attributed to both optic nerve infiltration along with raised ICP, as there were also presence of large peri-papillary hemorrhages. Tamoxifen therapy is known to cause raised intracranial pressure and optic disc swelling. However in our case, CSF cytology showed the presence of malignant cells and a drastic drop in vision which also excluded the possibility of idiopathic intracranial pressure caused by tamoxifen.

Both the response of LC to systemic or intrathecal chemotherapy and response of optic nerve metastasis to radiotherapy are variable. Outcome of these conditions are relatively poor. The median survival of patients who develop LC is four months in spite of optimum treatment [6]. Further prospective trials are necessary to determine the efficacy of available treatment modalities on overall survival and quality of life.

## Conclusions

The diagnosis of LC is based on three facets: the detection of malignant cells in the CSF, the demonstration of findings consistent with LC on MRI, and the presence of new clinical neurologic signs and symptoms. Diagnosis during the early stages presents a significant challenge whereby the neuroimaging may appear normal and the lumbar puncture carries a high false negative result. Due to this, experts have suggested repeated and multiple high-volume taps for CSF cytology if clinical suspicion of LC is high. Early diagnosis and multidisciplinary approach are crucial to improve treatment outcomes and quality of life.
